# Identifying the target population and preventive strategies to combat feline obesity

**DOI:** 10.1177/1098612X241228042

**Published:** 2024-02-28

**Authors:** Hannah Godfrey, Shawna Morrow, Sarah K Abood, Adronie Verbrugghe

**Affiliations:** 1 Department of Biomedical Sciences, Ontario Veterinary College, University of Guelph, Guelph, ON, Canada; 2 Department of Clinical Studies, Ontario Veterinary College, University of Guelph, Guelph, ON, Canada

**Keywords:** Weight gain, nutrition, energy requirement, gonadectomy, feeding management, dietary intervention

## Abstract

Feline obesity continues to be a priority health and welfare issue. Most research surrounding obesity currently focuses on obesity treatment. However, treatment for feline obesity is slow, often unsuccessful and not without consequences. Identifying high-risk populations for obesity onset is crucial for developing and implementing preventive strategies. This review identifies post-gonadectomy kittens aged 5–12 months as the primary target population for obesity prevention in domestic cats and highlights dietary and feeding management strategies to be implemented for obesity prevention.

## Importance of obesity prevention

Feline obesity affects 11–63% of domestic cats^1–5^ and is considered a health and welfare priority.^6^ Obesity is characterized as excess adipose tissue accumulation that can result in negative health consequences.^7,8^ For cats, these consequences can include, but are not limited to, insulin resistance, diabetes mellitus, osteoarthritis and skin conditions.^4,8–10^

Obesity treatment and management is a slow, often unsuccessful, process that can include energy restriction, veterinary therapeutic diets for weight loss, feeding management strategies and exercise for cats.^11^ Various treatment and management plans as well as risks for obesity and weight loss plans for cats have been reviewed extensively elsewhere.^11,12^ Poor owner compliance can be a setback for weight loss, possibly resulting from financial constraints, obesity or body condition score (BCS) misperceptions, or the inconvenience of weight loss plans. In addition, concurrent disorders can occur during slow weight loss, such as consequences of obesity (ie, diabetes mellitus), or from too rapid weight loss (ie, feline hepatic lipidosis), and can complicate the weight loss plan. Moreover, calorie restriction without an appropriately formulated diet can lead to nutrient deficiencies, specifically selenium and choline.^13^ Weight regain after treatment is all too common.^14^ In cats, it was found that weight regain after successful weight loss to an ideal weight resulted in higher body fat mass than during the original obese phase in all cats within an average of 14 weeks. Further, after successful weight loss by calorie restriction, more than half the cats regained over half the weight they had lost in a long-term follow-up (median of 954 days).^14^

While treatment has been a research priority for feline obesity, it is crucial that prevention strategies are communicated and implemented throughout a cat’s life span. Cat ownership is increasing steadily; thus, the number of cats requiring obesity management is likely to increase.^15,16^ As domestic cat populations continue to rise, a shift to focus on prevention becomes more critical.

The benefits of prevention are plentiful. In 2009, a report estimated that the first 12 months after a cat is diagnosed with obesity costs the pet owner over US$1000 in veterinary bills, without accounting for the additional costs of other veterinary care, such as clinical pathology charges and overnight hospital fees, or for any other health consequences.^17,18^ More recent reports state that owners of overweight or obese cats spend 36% more on diagnostic services and 53% more on surgical procedures than owners of normal-weight cats.^18,19^ Obesity is the most common nutritional disorder in cats within general clinical practice.^7,12^ Reducing the strain on the veterinary community can be achieved by reducing the number of cats that become obese and require this veterinary care. In addition, obesity affects welfare and quality of life.^6,20^ Due to the associated disorders that can arise from obesity,^7,21^ it is thought that obesity could also reduce the life span of cats, as has been found in dogs;^22,23^ however, this research does not yet exist for cats. To maintain and improve upon these variables, preventing obesity is a priority.

Major risk factors for feline obesity include age,^24,25^ sex,^25,26^ breed,^26^ indoor confinement,^26,27^ feeding primarily a dry food diet,^26–28^ free-feeding and feeding frequency,^29^ overestimating food allotments,^28,30^ owner misperceptions and even the human–animal bond.^31^ Interestingly, gonadectomy is consistently reported as a major risk factor for feline obesity (Table 1).^32–45^

**Table 1:** Summary of reported effects of gonadectomy on BW, FM, LSTM, EI, MER and EE in experiments with domestic cats

Reference	n	Sex	Life stage	Feeding method	BW	FM	LSTM	EI	MER	EE
Allaway et al, 2017^36^	16	Male	Growth	Stable BCS	Increased	N/A	N/A	Increased	N/A	NC
Backus et al, 2007^32^	24	Mixed	Growth	Ad lib	Increased	Increased	NC	Increased	N/A	N/A
Belsito et al, 2009^39^	8	Female	Adult	Ad lib*	Increased	Increased	N/A	Increased	N/A	N/A
Fettman et al, 1997^33^	12	Mixed	Adult	Ad lib	Increased	Increased	N/A	Increased	N/A	N/A
Flynn et al, 1996^40^	15	Female	Adult	Stable BW	N/A	N/A	N/A	N/A	Reduced	N/A
Harper et al, 2001^41^	49	Female	Adult	Ad lib	Increased	Increased	N/A	Increased	N/A	N/A
Hoenig & Ferguson, 2002^34^	20	Mixed	Adult	Stable BW	N/A	N/A	N/A	N/A	Reduced	N/A
Kanchuck et al, 2002^38^	32	Male	Adult	Ad lib	Increased	Increased	NC	Increased	N/A	N/A
Kanchuck et al, 2003^37^	16	Male	Adult	Ad lib	Increased	Increased	NC	Increased	N/A	NC
Martin et al, 2001^42^	42	Mixed	Adult	Ad lib	Increased	Increased	N/A	N/A	N/A	Reduced
Mitsuhashi et al, 2011^43^	22	Female	Adult	Pre-neuter MER	Increased	N/A	N/A	N/A	Reduced	N/A
Nguyen et al, 2004^44^	24	Mixed	Adult	Ad lib	Increased	Increased	N/A	NC	N/A	NC
Vester et al, 2009^45^	8	Female	Adult	Ad lib	Increased	Increased	N/A	Increased	N/A	Reduced
Wei et al, 2014^35^	9	Male	Adult	Ad lib	Increased	Increased	NC	Increased	N/A	NC

* Ad libitum feeding began 12 weeks after spaying; during weeks 0–12, cats were fed to maintain BW

Ad lib = ad libitum; BCS = body condition score; BW = body weight; EE = energy expenditure; EI = energy intake; FM = fat mass; LSTM = lean soft tissue mass; MER = maintenance energy requirements; N/A = not analyzed; NC = no change

## Role of gonadectomy in feline obesity

Previous reports estimate that over 80% of cats in North America and up to 92% of cats in the United Kingdom are gonadectomized.^46–49^ While gonadectomy increases the risk for obesity, there are also many benefits to this procedure, such as aiding in population control, reduced likelihood of abandonment, curbing negative behavioral patterns, and preventing certain diseases and reproductive disorders, such as mammary gland neoplasia, as previously described.^50^

Although many benefits are associated with gonadectomy, its role in obesity onset is alarming. Cats after gonadectomy have increased food intake, resulting in rapid body weight (BW) gain largely driven by increased body fat mass (Table 1).^32,33,35,37,45,51^ Comparison of the growth curves of neutered kittens with those of intact kittens further confirms growth disturbances, characterized by greater BW and body fat mass, following gonadectomy,^51^ and female kittens appear to be most affected. In addition, lower energy expenditure has been observed in cats after the gonadectomy procedure.^42,45^ Energy requirements can also be reduced by up to 30% after gonadectomy,^42^ while energy intake after gonadectomy is reported to increase by up to 50%.^32^ Several hypotheses for these findings have been proposed, such as reduced sexual hormone production affecting satiety hormones and growth hormones, and also a reduction in the energy required to produce and maintain sexual hormones and sex organs, though it is likely that this phenomenon is multifactorial, including a combination of hyperphagia, reduced physical activity and lowered energy requirements.^32,35,39,42,52^

The timing of neutering may influence this relationship in cats. The traditional age of neutering is often 6–9 months, whereas early-age neutering is considered at less than 5.5 months of age. Early-age neutering is still controversial; however, neutering and anesthesia procedures are considered safe for cats aged as young as 7 weeks.^53^ Various organizations recommend that cats be neutered at 6–14 weeks of age, or before 5 months.^53,54^ Early- and traditional-age spaying of female kittens were both found to require subsequent energy restriction to maintain ideal BCS;^36^ however, early-age spaying did not appear to induce acute hyperphagia, which was observed with traditional-age spaying. A recent investigation suggests that female kittens spayed early are more at risk of greater weight gain than female kittens spayed later in life.^51^ Regardless of sex, gonadectomy at both early and traditional age appears to increase the risk of weight gain resulting in obese conditions. Therefore, this population should be considered a primary target for obesity prevention.

## Growth as a target population for obesity prevention

Early development has previously been identified as a key life stage for preventing various diseases and disorders, such as obesity, in cats.^55^ In humans, poor nutrition during fetal development and childhood overweight or obese condition are associated with health complications into adulthood, such as obesity and diabetes mellitus; this is known as the Barker hypothesis.^56–60^ This hypothesis identifies that preventing and treating childhood obesity is essential to reducing obesity risks and prevalence in adulthood. These studies were conducted in humans; however, similar results have been observed in cats,^8,24,55,61–63^ such that rapid growth in kittens was a predictor of obesity in the adult life stage.^24^

Feline growth can be broken down into five stages (Table 2).^64–66^ To avoid interfering with skeletal growth, prevention should begin in the sustained growth phase. However, diet and feeding management strategies can be implemented in the post-weaning phase, particularly when early-age neutering occurs.

**Table 2:** The risk for obesity onset at the stages of growth for kittens^55,64–66^

		Growth phase	Age	Dietary habits	Expected growth rate	Description
Obesity prevention timeline and status	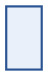	Neonatal	0–4 weeks	Rely on mother’s milk		More than 90% of time spent sleeping
	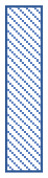	Weaning	4–8 weeks	Transition to solid food for growth	10–30 g per week	Increased time spent interacting with littermates; skill and behavior development; growth increases; reaching up to 100 g/week
	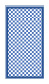	Post-weaning (rapid growth)	2–4 months	Food for growth, transitioned fully	<100 g per week	Regular vaccinations; early-age neutering
	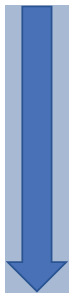	Sustained growth	5–12 months	Food for growth	Male: <20 g/day; Female: <11 g/day (80% of adult weight by the age of 8 months)	Energy requirements decrease; traditional-age neutering (6 months)
		Adulthood	12+ months	Transition to adult maintenance food	Skeletal maturity reached by 10–12 months	Additional growth for maturation and muscle development may occur up to 15 months
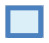 Minimal risk: weight monitored weekly to ensure appropriate growth						
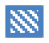 Minimal risk: weight monitored bi-weekly						
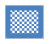 Moderate risk: weight monitored bi-weekly; food allotments weighed to energy requirements						
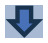 Critical risk: weight monitored weekly; implement and maintain obesity prevention strategies						

## Obesity prevention strategies for kittens

For kittens, multiple evidence-based strategies can be implemented to prevent obesity. A successful prevention plan involves commitment and compliance from the owner, and guidance from the veterinary team.^11,67^ Although further research is necessary to understand the influence of different communication strategies on the health outcome of pets, effective communication between the owner and the veterinary team is likely to have a positive impact on feline health. Meaningful discussion surrounding weight management could also play a key role in ensuring the prevention of obesity during the growth phase, and thereafter into adulthood.^68,69^ Additional strategies that can be implemented by the owner, including regular monitoring of growth and appropriate feeding practices, can also aid in successful obesity prevention.

### Effective veterinary–owner communication

The quality of communication can directly impact the strength of the veterinarian–pet owner bond, and, as a result, the standard of care the pet receives.^70,71^ By practicing effective communication skills, veterinarians can strengthen their bond with cat owners and improve client loyalty. This, in turn, can increase owners’ willingness to adhere to recommendations and, subsequently, improve upon clinical outcomes.^71–74^

Despite knowledge that communication is a key component in successful veterinary intervention, veterinary team members and pet owners inconsistently address the management and prevention of obesity during appointments.^67,69,75,76^ Reported barriers for veterinarians to discussing obesity prevention include concern that owners will be offended and/or non-compliant, fear of harming relationships with clients and of being seen as purely financially motivated, time constraints and lack of education on obesity management strategies.^68,70,77,78^ Barriers for pet owners include ineffective communication, in which the owner is made to feel judged or blamed and can result in defensiveness and less adherence to weight care.^78^

In an observational study using veterinarian and pet owner focus groups, owners said they would be more willing to adhere to recommendations if veterinarians take into consideration their lifestyle and their pet’s lifestyle, provide a variety of strategies, such as different diet choices and exercise regimes, and explain all aspects in a clear and direct manner rather than make ambiguous suggestions.^78^ Further, owners believed weight to be a vital part of their pet’s overall health, and that it should be measured and discussed at most appointments.^78^ However, observational data suggest that obesity prevention is discussed by veterinarians in fewer than 25% of appointments, and the degree to which it is discussed varies.^69^ For cat owners specifically, discussions surrounding a nutritional history of the cat, dietary recommendations, and ways to measure and maintain BCS are often incomplete or unclear.^69^ Without clear guidance from veterinarians when assessing appropriate BCS and diet, owners lack the proper tools and knowledge to effectively manage their cat’s weight. This has been seen in previous studies, in which cat owners misuse BCS charts, and incorrectly estimate their overweight or obese pet to be at an appropriate weight.^79,80^

To the authors’ knowledge, research focusing on weight management or obesity prevention communication from veterinary staff to new kitten owners has yet to be conducted. Current guidelines from the American Animal Hospital Association (AAHA) and American Association of Feline Practitioners recommend discussions surrounding diet and feeding practices begin at the kitten stage (up to 1 year), and discussions surrounding obesity risks and prevention strategies begin at the young adult stage (1–6 years).^81^ However, considering the high risk of obesity onset during growth, research investigating the impact of discussing obesity prevention with all kitten owners could prove beneficial.

If approached in a constructive and non-confrontational way, there is potential to have educational and effective conversations regarding obesity prevention between cat owners and veterinarians. When using effective communication strategies, obesity management can be developed into a long-term team effort, rather than an unsolicited or overwhelming intervention. Strategies to broach the topic of obesity prevention are summarized in Table 3.

**Table 3:** Effective communication strategies for veterinary professionals when discussing obesity prevention with cat owners

Strategies for effective communication	Ways to implement strategies	Justification
Utilize all members of the veterinary team	• Delegate aspects of communication to trained technicians or other veterinary support staff	Utilizing the knowledge and skill sets of all staff members in the practice can help provide pet owners with a variety of perspectives, suggestions and techniques for preventing obesity in their cat, and can maximize staff members’ time
Consider using the relationship-centered approach	• Treat relationships with pet owners as a partnership • Recognize and understand the client’s perspectives, motivations and expertise^82^ • Avoid authoritarian language, judgment and blame • Provide individualized, financially accessible, prevention strategies	Including owners in the decision-making process and ensuring their opinions are valued can lead to increased adherence to care^69,78^
Begin discussion at the first veterinary appointment for any cat owner, though specifically for owners of kittens, regardless of growth stage	• Provide/explain a BCS/growth chart • Include obesity prevention strategies in a kitten package or other brochures • Introduce ways to increase physical activity and enrichment (interactive feeding toys, dedicated play times)	Proper communication early on can promote obesity awareness to the cat owner and aid in preparations for prevention^11,68,69,83,84^
Encourage regular veterinary checkups for cat owners	• Encouragement can include: kitten’s improved behavior, specifically when at the clinic; early detection of diseases or disorders (reducing long-term costs and improving overall welfare); maintaining appropriate vaccination status; and an improved relationship between the veterinary team and owner^68,85,86^	Explaining the benefits of regular veterinary visits during the cat’s growth stage can encourage owners to schedule more frequent visits, allowing for greater maintenance of a weight management plan

BCS = body condition score

### Monitoring growth

One way to start the conversation in the consultation room is by use of kitten growth charts^66^ as part of an obesity prevention plan. This discussion should also encourage easy access to and use of a scale so owners can regularly weigh their kitten at home and plot its weight on the growth chart. Growth charts allow the owner and veterinary team to monitor growth and to identify any growth disturbances.^84^ Further, incorporating growth charts can foster the human–animal bond and owners may feel more involved in their kitten’s growth and development.

**Figure 1:**
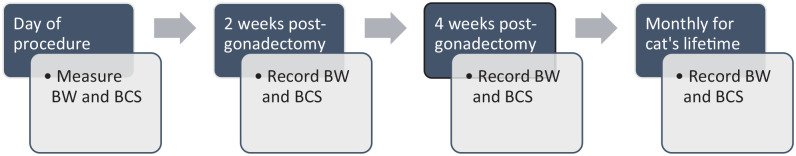
Recommended timeline for monitoring body weight (BW) and body condition score (BCS) during growth in kittens to prevent obesity after gonadectomy

For gonadectomized kittens, growth charts can be especially important for identifying impacts on BW (Figure 1). Plotting weight change on a graph, and teaching owners to do this at home, allows for identification of rapid or inappropriate weight gain after neutering. BCS and muscle condition score (MCS) can also be documented as a guide for body fat percentage; however, it is important to note that BCS and MCS, while validated for adult cats,^87,88^ are not validated for growth. Regardless, teaching cat owners how to BCS and MCS early in life can be beneficial in obesity prevention as obesity diagnosis in clinical practice uses BW, BCS, MCS and other morphometric measures, such as girth circumference.^11,85^

If rapid weight gain occurs, adjustments to the obesity prevention plan should be made and BW reassessed after 2 weeks.^84^ Further adjustments should be made until the rate of growth is back on track. Adjustments can also be made using feeding management strategies or nutritional interventions.

### Feeding to energy requirements

During growth, energy restriction is not recommended – rather, the prevention of additional excess weight gain is encouraged; therefore, determining and feeding to a kitten’s energy requirement rather than free-feeding is critical as an obesity prevention strategy.^11,84,89^ The gold standard for determining energy requirements is via indirect calorimetry; however, this is not available in a clinical setting.^90,91^ Therefore, for pet owners, reliance on predictive equations – BW, BCS and MCS monitoring, growth curves and individual progress, diet history and energy intake – is required.

Many predictive equations are proposed and available (Table 4). With regard to growth energy requirements, there can be differences between both the rapid and sustained growth phases and the appropriate calculations. Gross et al^64^ and AAHA^83^ use the traditional resting energy requirement and multiply by an appropriate life stage factor to calculate the daily energy requirement (DER). Alternatively, the National Research Council recommends one equation throughout both growth stages that incorporates the current BW of the kitten and the expected BW at maturity.^92^

**Table 4:** Published predictive equations to determine daily energy requirements (kcal/day) for growth in cats

Sex	Growth stage	Equation	Reference	Example* (kcal/d)
N/A	Rapid	DER (kcal/day) = (70 × BW^0.75^) × 3	^64^	353.20
N/A	Sustained	DER (kcal/day) = (70 × BW^0.75^) × 2.5	^64^	294.30
N/A	Rapid and sustained	DER (kcal/day) = (70 × BW^0.75^) × 2.5	^11^	294.30
N/A	Rapid and sustained	DER (kcal/day) = 100 × BW_a_^0.67^ × 6.7 × [e^(-0.189*p*)^-0.66]^†^	^92^	266.32
Male	Rapid and sustained	MEI (kcal/kg BW^0.67^/day) = 176.27^-0.037t^, R^2^ = 0.79^‡^	^93^	241.80
Female	Rapid and sustained	MEI (kcal/kg BW^0.67^/day) = 166.86^-0.044t^, R^2^ = 0.62‡	^93^	222.80

* Example using a 4-month-old kitten weighing 2.0 kg, and expected mature weight of 4 kg

† Where *p* = BWa/BWm, BW_a_/BW_m_; BW__a__ = actual body weight; BW_*_m_*_ = mature body weight; e = base of natural log ~2.718

‡ Where t is age in months

BW = body weight; DER = daily energy requirements; MEI = metabolizable energy intake

Expected mature BW can be estimated using published data on average BW for adult cats or by using growth charts developed from clinical data available for sexually intact kittens.^64,66,93^ While these equations can provide an estimate for the energy required, they do not account for sex or age or body composition. Merenda et al^93^ propose predictive equations for kittens that use age (in months) and sex to determine the energy requirements for growth (Table 4). These models account for both growth phases and are more specific to growth patterns in females and males, respectively. However, current equations and the proposed equations from Merenda et al^93^ are derived from research in colony cat populations using domestic shorthair cats. Research is not available for other breeds such as Maine Coon, Ragdoll and Sphynx. Further, data using client-owned cats for determining energy requirements via gold-standard methodologies are not available due to various limitations, such as acclimation to equipment, procedures and accessibility.^94^ Research methods for client-owned cats are limited to measuring the energy intake required to maintain stable BW or BCS, though this research can be valuable in making comparisons with colony cats and for determining models for energy requirements. More research can contribute to the understanding of cats’ energy requirements, specifically post-gonadectomy, as there are no equations currently available for cats after gonadectomy although research suggests a reduction in DER after gonadectomy.^42^

Discussing energy requirements and the calculations for food allotments for the cat with the pet owner is an important component to the growth and obesity prevention plan and should include guidelines on when, and how, to adjust food allotments based on BW and BCS. Calculating the food allotment is a simple equation:

$$Food\;Allotment\;\left(\frac{g}{day}\right)=DER\left(\frac{kcal}{day}\right)÷energy\;density\;(\frac{kcal}{g})$$

The most accurate way to measure the food allotment is by using a gram scale, and not by scoop or cup.^30,95^ Encouraging pet owners to use a gram scale and demonstrating how to properly use the scale can be an effective component in the prevention of obesity.

### Feeding management strategies

Many feeding management strategies can be used and implemented as part of the obesity prevention plan for kittens. As stated previously, kittens should be fed a food allotment that meets their DER and not free-fed.^11,85^ In addition, for single-cat homes, individualized feeders could reduce food-seeking behavior. For cats in multiple-pet households, microchip feeders as well as separating pets at mealtimes can reduce competition for food and overconsumption.^96–98^ A recent consensus statement recommends feeding cats multiple small meals per day to mitigate behavioral concerns and potentially improve welfare;^97^ however, once-a-day feeding may be beneficial in promoting satiety and fatty acid oxidation in cats compared with multiple feedings.^29^ To date, there is a dearth of data investigating the long-term physiological effects of feeding frequency in cats and its role in obesity prevention. Eliminating table scraps and minimizing treats to account for up to 10% of the DER is also an effective method to reduce excess calories being consumed.^11,12,85,97^

Food toys can increase physical activity and may have cognitive and enrichment benefits,^11,85,97^ though a recent pilot study found no effect of food toys on overall activity in adult neutered cats but found potential benefits for reducing stress and improving overall wellbeing.^99^ Moving or hiding food around the house can increase animal movement and provide enrichment as a way to mimic hunting for prey. Increasing exercise is often overlooked in cats but should always be encouraged as part of a lifestyle or obesity prevention plan.^11,84,85,89^ Dedicating a daily minimum of 15 minutes to playing with toys or the use of electronic or interactive toys provides regular daily activity, improves the human–animal bond and provides additional enrichment.^100,101^ This time can be broken up into small intervals throughout the day based on the owner’s schedule and flexibility. Vertical space is another way to increase physical activity for cats. Vertical space can be improved by cat trees, hammocks or shelves that encourage climbing behaviors.

### Nutritional interventions

Regardless of growth stage and BCS, kittens should be offered a life-stage-appropriate food. For kittens younger than 12 months, this should be a food labelled for growth or all life stages. Growth diets tend to have higher energy densities to meet the greater energy demands for growth, while also accounting for the small stomach capacity of kittens to prevent ‘gut-fill’.^64,65,102^ However, because of the high energy density, it is especially important to feed to DER, as a small increase in food amount can be a large increase in energy intake, resulting in excess weight gain.

If a kitten’s BCS is higher than ideal, owners should be discouraged from changing the diet to a weight management or weight loss option. Most weight management and weight loss foods do not meet the energy density demands for growth or the additional essential micronutrients required at higher levels for growth. Rather, veterinary-pet-owner communication is a priority to ensure emphasis on the importance of feeding to DER, adjusting as per individual needs. In addition, utilizing the feeding management practices detailed in this review should be encouraged.

#### Macronutrients

Dietary protein, fat and carbohydrates, but not dietary fiber, contribute to the energy density of food. Regardless, each macronutrient has a potential role in obesity prevention (Table 5).^92^

**Table 5:** Roles for macronutrients in the diet of kittens and the recommendations and dietary targets for these in obesity prevention^11,64,85,92,103^

	Role in growth	Recommendations for obesity prevention	Target
Energy	Higher energy density for increased demand during growth; higher energy density reduces food amount required and accounts for limited expandability of kitten stomachs	Encourage feeding to DER; look for lower energy density growth diets	4000–4500 kcal/kg ME
Protein	Delivers nitrogen and amino acids; growth and maintenance of muscle mass; immune system and function; enzyme and hormones; structural components	High-protein diets are beneficial for growth and muscle mass	40–45% crude protein DM
Fat	Delivers essential fatty acids during growth (linoleic acid, arachidonic acid, a-linolenic acid, EPA and DHA); vehicle for absorption of fat-soluble vitamins; energy dense; improves palatability	Moderate fat levels for energy density and palatability; lower fat content will be found in lower energy density growth diets	18–35% crude fat DM
Carbohydrates	No dietary requirement though glucose is physiologically essential; contributes to energy density; important for texture and structure of food	NFE estimates carbohydrates; minimize simple sugars; focus on complex carbohydrates	12–37% DM*
Dietary fiber	No dietary requirement; beneficial for gut motility, building/feeding the microbiome and providing bulk in the gastrointestinal tract; crude fiber only accounts for insoluble fibers, not soluble fibers	Amount and types are relatively unknown for cats for obesity prevention	5–8% DM

* Calculated based on lower and upper ranges of additional macronutrients

DER = daily energy requirement; DHA = docosahexaenoic acid; DM = dry matter; EPA = eicosapentaenoic acid; ME = metabolizable energy; NFE = nitrogen-free extract

Limited research has been conducted on the effects of macronutrient compositions after gonadectomy; however, consistent with previous reports in adult cats without gonadectomy as a factor,^104,105^ gonadectomized cats of both sexes experience a positive correlation between BW and fat mass with increasing dietary fat content.^32,44^ In both studies, cats were gonadectomized at 7–10 months of age, they were fed ad libitum and test diets were not formulated specifically for growth. Overall, the results of these studies suggest that high-fat diets may present a challenge for preventing obesity after gonadectomy, though more research is required. Currently, there are no studies, to the authors’ knowledge, assessing dietary macronutrients in obesity prevention during growth, specifically after gonadectomy.

Dietary fiber is often added to diets for the management of obesity due to its proposed role in diluting energy density, reducing overall energy intake and its effects on gastric emptying, satiety and promoting a healthy microbiome.^106–110^ Regarding obesity prevention, the effects of fiber on satiety could be beneficial for cats when fed ad libitum (as commonly occurs with kittens). Fiber has shown benefits in weight loss, diabetes and gastrointestinal diseases in cats.^109,111–115^ Research on fiber for obesity prevention in cats is lacking, specifically after gonadectomy and during growth.

#### Diet format

Often overlooked is the format of the diet. Of particular interest, when fed ad libitum for 4 weeks after gonadectomy, cats consuming an extruded dry food were reported to have greater weight gain and increased BCS at weeks 5 and 6 after neutering compared with cats consuming a wet canned food.^116^ These findings were likely attributed to the lower energy density of the wet food compared with the dry food. Further, it was also reported that adding 40% water to a dry food improved physical activity levels and weight gain was lower despite similar energy intake compared with a control group.^117^ Thus, moisture content and proper hydration may have an important role in weight management. To the authors’ knowledge, research investigating different diet formats for obesity prevention after gonadectomy is lacking. This is particularly important due to the rapid growth of the pet food industry and the introduction of various food formats from extruded kibble and wet foods to gently cooked and raw meat-based diets, which can add another complexity to nutritional interventions.^118^

## Conclusions

Recognizing the significance of early life nutrition and growth, specifically after neutering in obesity prevention is paramount. The Barker hypothesis, supported by subsequent studies in humans and cats alike, underscores the lasting impact of early prevention of obesity on long-term health. For kittens, a comprehensive approach is essential, involving effective communication between veterinary teams and owners, vigilant and thorough growth monitoring and prevention plans, and optimal feeding management strategies that can include tailored diets, macronutrient balance and environmental enrichment. By prioritizing these measures, veterinary teams and owners can positively influence the lifelong health of cats, specifically the vital role of early interventions in curbing the long-term effects of obesity.

## References

[1] Michel K Scherk M. From problem to success: feline weight loss programs that work. J Feline Med Surg 2012; 14: 327–336. 22511475 10.1177/1098612X12444999 PMC11132260

[bibr2-1098612X241228042] Courcier EA O’Higgins R Mellor DJ , et al. Prevalence and risk factors for feline obesity in a first opinion practice in Glasgow, Scotland. J Feline Med Surg 2010; 12: 746–753. 20685143 10.1016/j.jfms.2010.05.011 PMC11135528

[bibr3-1098612X241228042] Colliard L Paragon BM Lemuet B , et al. Prevalence and risk factors of obesity in an urban population of healthy cats. J Feline Med Surg 2009; 11: 135–140. 18774325 10.1016/j.jfms.2008.07.002 PMC10832791

[bibr4-1098612X241228042] Tarkosova D Story MM Rand JS , et al. Feline obesity – prevalence, risk factors, pathogenesis, associated conditions and assessment: a review. Veterinární Medicína 2016; 61: 295–307.

[5] Mendes-Junior AF Passos CB Gáleas MAV , et al. Prevalence and risk factors of feline obesity in Alegre, Espírito Santo, Brazil. Semina Ciênc Agrár 2013; 34: 1801–1806.

[6] Rioja-Lang F Bacon H Connor M , et al. Determining priority welfare issues for cats in the United Kingdom using expert consensus. Vet Rec Open 2019; 6. DOI: 10.1136/vetreco-2019-000365. PMC6861065 31798909

[7] German AJ . The growing problem of obesity in dogs and cats. J Nutr 2006; 136 Suppl 7: 1940–1946. 10.1093/jn/136.7.1940S 16772464

[8] Lund EM Armstrong PJ Kirk CA , et al. Prevalence and risk factors for obesity in adult cats from private US veterinary practices. Intern J Appl Res Vet Med 2005; 3: 88–96.

[bibr9-1098612X241228042] Rand JS Marshall RD. Diabetes mellitus in cats. Vet Clin North Am Small Anim Pract 2005; 35: 211–224. 15627634 10.1016/j.cvsm.2004.10.001

[10] Scarlett JM Donoghue S . Associations between body condition and disease in cats. J Am Vet Med Assoc 1998; 212: 1725–1731. 9621878

[11] Cline MG Burns KM Coe JB , et al. 2021 AAHA nutrition and weight management guidelines for dogs and cats. J Am Anim Hosp Assoc 2021; 57: 153–178. 34228790 10.5326/JAAHA-MS-7232

[12] Laflamme DP. Understanding and managing obesity in dogs and cats. Vet Clin North Am Small Anim Pract 2006; 36: 1283–1295. 17085235 10.1016/j.cvsm.2006.08.005

[13] Grant CE Shoveller AK Blois S , et al. Dietary intake of amino acids and vitamins compared to NRC requirements in obese cats undergoing energy restriction for weight loss. BMC Vet Res 2020; 16: 426. DOI: 10.1186/s12917-020-02649-0. 33160364 PMC7648986

[14] Deagle G Holden SL Biourge V , et al. Long-term follow-up after weight management in obese cats. J Nutr Sci 2014; 3: e25. DOI: 10.1017/jns.2014.36. PMC4473167 26101594

[15] Agriculture Canada. Sector trend analysis – pet food trends in Canada. https://agriculture.canada.ca/en/international-trade/market-intelligence/reports/sector-trend-analysis-pet-food-trends-canada (2021, accessed 30 June 2023).

[16] American Pet Products Association. Pet industry market size, trends & ownership statistics. https://www.americanpetproducts.org/ (accessed 07 April 2023).

[17] Bartlett P Van Buren J. Counting the cost of chronic disease. https://www.dvm360.com/view/counting-cost-chronic-disease (2009, accessed 30 June 2023).

[18] Bomberg E Birch L Endenburg N , et al. The financial costs, behaviour and psychology of obesity: a one health analysis. J Comp Pathol 2017; 156: 310–325. 28460796 10.1016/j.jcpa.2017.03.007

[19] Banfield Pet Hospital. State of pet health®. https://www.banfield.com/pet-health/State-of-pet- (accessed 07 April 2023).

[20] Hanford R Linder DE . Impact of obesity on quality of life and owner’s perception of weight loss programs in cats. Vet Sci 2021; 8. DOI: 10.3390/vetsci8020032. PMC7924056 33672603

[21] Sallander M Eliasson J Hedhammar Å . Prevalence and risk factors for the development of diabetes mellitus in Swedish cats. Acta Vet Scand 2012; 54. DOI: 10.1186/1751-0147-54-61. PMC3537597 23114390

[22] Kealy RD Lawler DF Ballam JM , et al. Effects of diet restriction on life span and age-related changes in dogs. J Am Vet Med Assoc 2002; 220: 1315–1320. 11991408 10.2460/javma.2002.220.1315

[23] Salt C Morris PJ Wilson D , et al. Association between life span and body condition in neutered client-owned dogs. J Vet Intern Med 2019; 33: 89–99. 30548336 10.1111/jvim.15367 PMC6335446

[24] Serisier S Feugier A Venet C , et al. Faster growth rate in ad libitum-fed cats: a risk factor predicting the likelihood of becoming overweight during adulthood. J Nutr Sci 2013; 2: e11. DOI: 10.1017/jns.2013.10. PMC4153074 25191559

[25] Courcier EA Mellor DJ Pendlebury E , et al. An investigation into the epidemiology of feline obesity in Great Britain: results of a cross-sectional study of 47 companion animal practises. Vet Rec 2012; 171: 560. DOI: 10.1136/vr.100953. 23081976

[26] Teng KT McGreevy PD Toribio JLML , et al. Risk factors for underweight and overweight in cats in metropolitan Sydney, Australia. Prev Vet Med 2017; 144: 102–111. 28716190 10.1016/j.prevetmed.2017.05.021

[bibr27-1098612X241228042] Rowe E Browne W Casey R , et al. Risk factors identified for owner-reported feline obesity at around one year of age: dry diet and indoor lifestyle. Prev Vet Med 2015; 121: 273–281. 26265631 10.1016/j.prevetmed.2015.07.011

[28] Rowe EC Browne WJ Casey RA , et al. Early-life risk factors identified for owner-reported feline overweight and obesity at around two years of age. Prev Vet Med 2017; 143: 39–48. 28622790 10.1016/j.prevetmed.2017.05.010

[29] Camara A Verbrugghe A Cargo-Froom C , et al. The daytime feeding frequency affects appetite-regulating hormones, amino acids, physical activity, and respiratory quotient, but not energy expenditure, in adult cats fed regimens for 21 days. PLoS One 2020; 15. DOI: 10.1371/journal.pone.0238522. PMC7500645 32946478

[30] German AJ Holden SL Mason SL , et al. Imprecision when using measuring cups to weigh out extruded dry kibbled food. J Anim Physiol Anim Nutr 2011; 95: 368–373. 10.1111/j.1439-0396.2010.01063.x 21039926

[31] Kienzle E Bergler R Mandernach A . A comparison of the feeding behavior and the human–animal relationship in owners of normal and obese dogs. J Nutr 1998; 128: 2779S–2782S. 10.1093/jn/128.12.2779S 9868265

[32] Backus RC Cave NJ Keisler DH . Gonadectomy and high dietary fat but not high dietary carbohydrate induce gains in body weight and fat of domestic cats. Br J Nutr 2007; 98: 641–650. 17524182 10.1017/S0007114507750869

[bibr33-1098612X241228042] Fettman MJ Stanton CA Banks LL , et al. Effects of neutering on bodyweight, metabolic rate and glucose tolerance of domestic cats. Res Vet Sci 1997; 62: 131–136. 9243711 10.1016/s0034-5288(97)90134-x

[bibr34-1098612X241228042] Hoenig M Ferguson DC . Effects of neutering on hormonal concentrations and energy requirements in male and female cats. Am J Vet Res 2002; 63: 634–639. 12013460 10.2460/ajvr.2002.63.634

[bibr35-1098612X241228042] Wei A Fascetti AJ Kim K , et al. Early effects of neutering on expenditure in adult male cats. PLoS One 2014; 9. DOI: 10.1371/journal.pone.0089557. PMC3935885 24586869

[bibr36-1098612X241228042] Allaway D Gilham M Colyer A , et al. The impact of time of neutering on weight gain and energy intake in female kittens. J Nutr Sci 2017; 6: e19. DOI: 10.1017/jns.2017.20. PMC5468748 28630696

[bibr37-1098612X241228042] Kanchuk ML Backus RC Calvert CC. Weight gain in normal and lipase–deficient male domestic cats results from increased food intake and not decreased energy expenditure. J Nutr 2003; 133: 1866–1874. 12771331 10.1093/jn/133.6.1866

[bibr38-1098612X241228042] Kanchuk ML Backus RC Calvert CC , et al. Neutering induces changes in food intake, body weight, plasma insulin and leptin concentrations in normal and lipoprotein lipase-deficient male cats. J Nutr 2002; 132: 1730–1732. 10.1093/jn/132.6.1730S 12042509

[bibr39-1098612X241228042] Belsito KR Vester BM Keel T , et al. Impact of ovariohysterectomy and food intake on body composition, physical activity, and adipose gene expression in cats. J Anim Sci 2009; 87: 594–602. 18997063 10.2527/jas.2008-0887

[bibr40-1098612X241228042] Flynn MF Hardie EM Armstrong PJ . Effect of ovariohysterectomy on maintenance energy requirement in cats. J Am Vet Med Assoc 1996; 209: 1572–1581. 8899020

[bibr41-1098612X241228042] Harper EJ Stack DM Watson TD , et al. Effects of feeding regimens on bodyweight, composition and condition score in cats following ovariohysterectomy. J Small Anim Pract 2001; 42: 433–438. 11570385 10.1111/j.1748-5827.2001.tb02496.x

[bibr42-1098612X241228042] Martin L Siliart B Dumon H , et al. Leptin, body fat content and energy expenditure in intact and gonadectomized adult cats: a preliminary study. J Anim Physiol Anim Nutr 2001; 85: 195–199. 10.1046/j.1439-0396.2001.00322.x 11686788

[bibr43-1098612X241228042] Mitsuhashi Y Chamberlin AJ Bigley KE , et al. Maintenance energy requirement determination of cats after spaying. Br J Nutr 2011; 106 Suppl 1: S135–138. 10.1017/S0007114511001899 22005410

[bibr44-1098612X241228042] Nguyen PG Dumon HJ Siliart BS , et al. Effects of dietary fat and energy on body weight and composition after gonadectomy in cats. Am J Vet Res 2004; 65: 1708–1713. 15631038 10.2460/ajvr.2004.65.1708

[45] Vester BM Sutter SM Keel TL , et al. Ovariohysterectomy alters body composition and adipose and skeletal muscle gene expression in cats fed a high-protein or moderate-protein diet. Animal 2009; 3: 1287–1298. 22444905 10.1017/S1751731109004868

[46] Perrin T. The Business of Urban Animals Survey: the facts and statistics on companion animals in Canada. Can Vet J 2009; 50: 48–52. 19337613 PMC2603652

[47] Chu K Anderson WM Rieser MY . Population characteristics and neuter status of cats living in households in the United States. J Am Vet Med Assoc 2009; 234: 1023–1030. 19366332 10.2460/javma.234.8.1023

[48] Trevejo R Yang M Lund EM . Epidemiology of surgical castration of dogs and cats in the United States. J Am Vet Med Assoc 2011; 238: 898–904. 21453178 10.2460/javma.238.7.898

[49] Murray JK Roberts MA Whitmars A , et al. Survey of the characteristics of cats owned by households in the UK and factors affecting their neutered status. Vet Rec 2009; 164: 137–141. 19188344 10.1136/vr.164.5.137

[50] Reichler IM . Gonadectomy in cats and dogs: a review of risks and benefits. Reprod Domest Anim Zuchthyg 2009; 44 Suppl 2: 29–35. 10.1111/j.1439-0531.2009.01437.x 19754532

[51] Salt C Butterwick R Henzel K , et al. Comparison of growth in neutered domestic shorthair kittens with growth in sexually-intact cats. PLoS One 2023; 18. DOI: 10.1371/journal.pone.0283016. PMC10016642 36920976

[52] Vendramini THA Amaral AR Pedrinelli V , et al. Neutering in dogs and cats: current scientific evidence and importance of adequate nutritional management. Nutr Res Rev 2020; 33: 134–144. 31931899 10.1017/S0954422419000271

[53] **AAFP Position Statement: pediatric sterilization in cats.** J Feline Med Surg 2020; 22: 870. DOI: 10.1177/1098612X20948325. PMC11135659 32845229

[54] Federation of Veterinarians of Europe. Early neutering of kittens. https://fve.org/publications/early-neutering-of-kittens/ (2019, accessed 28 August 2023).

[55] Gaillard V Chastant S England G , et al. Environmental risk factors in puppies and kittens for developing chronic disorders in adulthood: a call for research on developmental programming. Front Vet Sci 2022; 9. DOI: 10.3389/fvets.2022.944821. PMC9816871 36619947

[56] Reilly JJ Armstrong J Dorosty AR , et al. Early life risk factors for obesity in childhood: cohort study. BMJ 2005; 330: 1357. DOI: 10.1136/bmj.38470.670903.E0. PMC558282 15908441

[57] Barker DJP Eriksson JG Forsén T , et al. Fetal origins of adult disease: strength of effects and biological basis. Int J Epidemiol 2002; 31: 1235–1239. 12540728 10.1093/ije/31.6.1235

[58] Sun SS Liang R Huang TTK , et al. Childhood obesity predicts adult metabolic syndrome: the Fels Longitudinal Study. J Pediatr 2008; 152: 191–200. 18206688 10.1016/j.jpeds.2007.07.055 PMC3988700

[59] Liang Y Hou D Zhao X , et al. Childhood obesity affects adult metabolic syndrome and diabetes. Endocrine 2015; 50: 87–92. 25754912 10.1007/s12020-015-0560-7

[60] Umer A Kelley GA Cottrell LE , et al. Childhood obesity and adult cardiovascular disease risk factors: a systematic review with meta-analysis. BMC Public Health 2017; 17: 683. DOI: 10.1186/s12889-017-4691-z. 28851330 PMC5575877

[61] Linder D Mueller M. Pet obesity management: beyond nutrition. Vet Clin North Am Small Anim Pract 2014; 44: 789–806. 24951347 10.1016/j.cvsm.2014.03.004

[bibr62-1098612X241228042] Zoran DL Rand JS . The role of diet in the prevention and management of feline diabetes. Vet Clin North Am Small Anim Pract 2013; 43: 233–243. 23522169 10.1016/j.cvsm.2012.11.004

[63] Cave NJ Bridges JP Weidgraaf K , et al. Nonlinear mixed models of growth curves from domestic shorthair cats in a breeding colony, housed in a seasonal facility to predict obesity. J Anim Physiol Anim Nutr 2018; 102: 1390–1400. 10.1111/jpn.12930 29932481

[64] Gross KL Becvarova I Debraekeleer J. Feeding growing kittens: postweaning to adulthood. In: Hand MS Lewis LD (eds). Small animal clinical nutrition. 5th ed. Topeka: Mark Morris Institute, 2010, pp 429–436.

[bibr65-1098612X241228042] Case LP Daristotle L Hayek MG , et al. Canine and feline nutrition: a resource for companion animal professionals. 3rd ed. Toronto: Elsevier, 2010.

[66] Salt C German AJ Henzel KS , et al. Growth standard charts for monitoring bodyweight in intact domestic shorthair kittens from the USA. PLoS One 2022; 17. DOI: 10.1371/journal.pone.0277531. PMC9678321 36409712

[67] Sutherland KA Coe JB O’Sullivan TL . Exploring veterinary professionals’ perceptions of pet weight-related communication in companion animal veterinary practice. Vet Rec 2023; 192. DOI: 10.1002/vetr.1973. 35915963

[68] Churchill J Ward E. Communicating with pet owners about obesity. Vet Clin North Am Small Anim Pract 2016; 46: 899–911. 27264055 10.1016/j.cvsm.2016.04.010

[69] Phillips AM Coe JB Rock MJ , et al. Feline obesity in veterinary medicine: insights from a thematic analysis of communication in practice. Front Vet Sci 2017; 4. DOI: 10.3389/fvets.2017.00117. PMC5534443 28824925

[70] Lue TW Pantenburg DP Crawford PM . Impact of the owner-pet and client-veterinarian bond on the care that pets receive. J Am Vet Med Assoc 2008; 232: 531–540. 18279086 10.2460/javma.232.4.531

[71] Abood SK . Effectively communicating with your clients. Top Companion Anim Med 2008; 23: 143–147. 18656842 10.1053/j.tcam.2008.04.007

[72] Coe JB Adams CL Eva K , et al. Development and validation of an instrument for measuring appointment-specific client satisfaction in companion-animal practice. Prev Vet Med 2010; 93: 201–210. 19926150 10.1016/j.prevetmed.2009.10.005

[73] Dysart LMA Coe JB Adams CL . Analysis of solicitation of client concerns in companion animal practice. J Am Vet Med Assoc 2011; 238: 1609–1615. 21671816 10.2460/javma.238.12.1609

[74] Kanji N Coe JB Adams CL , et al. Effect of veterinarian-client-patient interactions on client adherence to dentistry and surgery recommendations in companion-animal practice. J Am Vet Med Assoc 2012; 240: 427–436. 22309015 10.2460/javma.240.4.427

[75] Sutherland KA Coe JB O’Sullivan TL. Assessing owners’ readiness to change their behaviour to address their companion animal’s obesity. Vet Rec 2023; 192. DOI: 10.1002/vetr.1979. 36073659

[76] Kipperman BS German AJ . The responsibility of veterinarians to address companion animal obesity. Animals 2018; 8. DOI: 10.3390/ani8090143. PMC6162666 30134516

[77] Bartges J Kushner RF Michel KE , et al. One health solutions to obesity in people and their pets. J Comp Pathol 2017; 156: 326–333. 28460797 10.1016/j.jcpa.2017.03.008

[78] Sutherland KA Coe JB Janke N , et al. Pet owners’ and companion animal veterinarians’ perceptions of weight-related veterinarian-client communication. J Am Vet Med Assoc 2022; 260: 1697–1703. 35905163 10.2460/javma.22.03.0101

[79] Peron L Rahal SC Castilho MS , et al. Owner’s perception for detecting feline body condition based on questionnaire and scores. Top Companion Anim Med 2016; 31: 122–124. 27968812 10.1053/j.tcam.2016.08.008

[80] Teixeira FA Queiroz MR Oba PM , et al. Brazilian owners perception of the body condition score of dogs and cats. BMC Vet Res 2020; 16: 463. DOI: 10.1186/s12917-020-02679-8. 33246455 PMC7694915

[81] Quimby J Gowland S Carney HC , et al. 2021 AAHA/AAFP feline life stage guidelines. J Feline Med Surg 2021; 23: 211–233. 33627003 10.1177/1098612X21993657 PMC10812130

[82] Shaw JR Bonnett BN Adams CL , et al. Veterinarian-client-patient communication patterns used during clinical appointments in companion animal practice. J Am Vet Med Assoc 2006; 228: 714–721. 16506932 10.2460/javma.228.5.714

[83] Baldwin K Bartges J Buffington T , et al. AAHA nutritional assessment guidelines for dogs and cats. J Am Anim Hosp Assoc 2010; 46: 285–296. 20610704 10.5326/0460285

[84] Shepherd M. Canine and feline obesity management. Vet Clin North Am Small Anim Pract 2021; 51: 653–667. 33653534 10.1016/j.cvsm.2021.01.005

[85] Freeman L Becvarova I Cave N , et al. WSAVA nutritional assessment guidelines. J Small Anim Pract 2011; 52: 385–396. 21649660 10.1111/j.1748-5827.2011.01079.x

[86] American Animal Hospital Association-American Veterinary Medical Association Preventive Healthcare Guidelines Task Force. Development of new canine and feline preventive healthcare guidelines designed to improve pet health. J Am Anim Hosp Assoc 2011; 47: 306–311. 21896837 10.5326/JAAHA-MS-4007

[87] Laflamme DP . Development and validation of a body condition score system for cats: a clinical tool. Feline Practice 1997; 25: 13–18.

[88] Michel KE Anderson W Cupp C , et al. Correlation of a feline muscle mass score with body composition determined by dual-energy X-ray absorptiometry. Br J Nutr 2011; 106: S57–59. 10.1017/S000711451100050X 22005437

[89] Cline MG Murphy M. Obesity in the dog and cat. 1st ed. Boca Raton: CRC Press, 2019.

[90] Levine JA . Measurement of energy expenditure. Public Health Nutr 2005; 8: 1123–1132. 16277824 10.1079/phn2005800

[91] Schoeller DA Cook CM Raman A. **Energy expenditure: indirect calorimetry.** In: Caballero B (ed). Encyclopedia of human nutrition. 3rd ed. Oxford: Elsevier, 2012, pp 170–176.

[92] National Research Council. Nutrient requirements of dogs and cats. Washington DC: The National Academies Press, 2006, pp 354–370.

[93] Merenda MEZ Sato J Scheibel S , et al. Growth curve and energy intake in male and female cats. Top Companion Anim Med 2021; 44. DOI: 10.1016/j.tcam.2021.100518. 33549804

[94] Gooding MA Duncan IJH Atkinson JL , et al. Development and validation of a behavioral acclimation protocol for cats to respiration chambers used for indirect calorimetry studies. J Appl Anim Welf Sci 2012; 15: 144–162. 22458875 10.1080/10888705.2012.658332

[95] Coe JB Rankovic A Edwards TR , et al. Dog owner’s accuracy measuring different volumes of dry dog food using three different measuring devices. Vet Rec 2019; 185: 599. DOI: 10.1136/vr.105319. 31409751 PMC6902066

[96] Witzel-Rollins A Murphy M Springer CM , et al. Evaluation of a pet-separating automatic feeder and high-frequency meal feeding for weight loss in multi-cat households. J Feline Med Surg 2022; 24: e281–e288. 10.1177/1098612X221105046 PMC10812286 35762268

[bibr97-1098612X241228042] Sadek T Hamper B Horwitz D , et al. Feline feeding programs: addressing behavioural needs to improve feline health and wellbeing. J Feline Med Surg 2018; 20: 1049–1055. 30375945 10.1177/1098612X18791877 PMC11343346

[98] Hadar BN Lambrecht KJ Poljak Z , et al. Technology-enhanced weight-loss program in multiple-cat households: a randomized controlled trial. J Feline Med Surg 2022; 24: 726–738. 34672236 10.1177/1098612X211044412 PMC9315194

[99] Naik R Witzel A Albright JD , et al. Pilot study evaluating the effect of feeding method on overall activity of neutered indoor pet cats. J Vet Behav 2018; 25: 9–13.

[100] Kienzle E Bergler R. Human-animal relationship of owners of normal and overweight cats. J Nutr 2006; 136: 1947S–1950S. 10.1093/jn/136.7.1947S 16772465

[101] Henning JSL Nielsen T Fernandez E , et al. Factors associated with play behavior in human-cat dyads. J Vet Behav 2022; 52–53: 21–30.

[102] Lawler DF . Neonatal and pediatric care of the puppy and kitten. Theriogenology 2008; 70: 384–392. 18513788 10.1016/j.theriogenology.2008.04.019

[103] Ha MA Jarvis MC Mann JI . A definition for dietary fibre. Eur J Clin Nutr 2000; 54: 861–864. 11114682 10.1038/sj.ejcn.1601109

[104] Wei A Fascetti AJ Liu KJ , et al. Influence of a high-protein diet on energy balance in obese cats allowed ad libitum access to food. J Anim Physiol Anim Nutr 2011; 95: 359–367. 10.1111/j.1439-0396.2010.01062.x 21039925

[105] Gooding MA Atkinson JL Duncan IJH , et al. Dietary fat and carbohydrate have different effects on body weight, energy expenditure, glucose homeostasis and behaviour in adult cats fed to energy requirement. J Nutr Sci 2015; 4: e2. DOI: 10.1017/jns.2014.60. PMC4463014 26090098

[106] Anderson JW Akanji AO. Dietary fiber – an overview. Diabetes Care 1991; 14: 1126–1131. 1663444 10.2337/diacare.14.12.1126

[bibr107-1098612X241228042] Butowski CF Thomas DG Cave NJ , et al. In vitro assessment of hydrolysed collagen fermentation using domestic cat (*Felis catus*) faecal inocula. Animals 2022; 12. DOI: 10.3390/ani12040498. PMC8868200 35203206

[bibr108-1098612X241228042] Butowski CF Thomas DG Young W , et al. Addition of plant dietary fibre to a raw red meat high protein, high fat diet, alters the faecal bacteriome and organic acid profiles of the domestic cat (*Felis catus*). PLoS One 2019; 14. DOI: 10.1371/journal.pone.0216072. PMC6493751 31042730

[bibr109-1098612X241228042] Fischer MM Kessler AM de Sá LRM , et al. Fiber fermentability effects on energy and macronutrient digestibility, fecal traits, postprandial metabolite responses, and colon histology of overweight cats. J Anim Sci 2012; 90: 2233–2245. 22247109 10.2527/jas.2011-4334

[110] Cline M Witzel AL Moyers T , et al. Comparison of high fiber and low carbohydrate diets on owner-perceived satiety of cats during weight loss. Am J Anim Vet Sci 2012; 7: 218–225.

[111] Bissot T Servet E Vidal S , et al. Novel dietary strategies can improve the outcome of weight loss programmes in obese client-owned cats. J Feline Med Surg 2010; 12: 104–112. 19682935 10.1016/j.jfms.2009.07.003 PMC10911441

[112] Bennett N Greco DS Peterson ME , et al. Comparison of a low carbohydrate–low fiber diet and a moderate carbohydrate–high fiber diet in the management of feline diabetes mellitus. J Feline Med Surg 2006; 8: 73–84. 16275041 10.1016/j.jfms.2005.08.004 PMC10832676

[113] Moreno AA Parker VJ Winston JA , et al. Dietary fiber aids in the management of canine and feline gastrointestinal disease. J Am Vet Med Assoc 2022; 260: S33–S45. 10.2460/javma.22.08.0351 36288203

[114] Nelson RW Scott-Moncrieff JC Feldman EC , et al. Effect of dietary insoluble fiber on control of glycemia in cats with naturally acquired diabetes mellitus. J Am Vet Med Assoc 2000; 216: 1082–1088. 10754667 10.2460/javma.2000.216.1082

[115] Pallotto MR Godoy MRC de Holscher HD , et al. Effects of weight loss with a moderate-protein, high-fiber diet on body composition, voluntary physical activity, and fecal microbiota of obese cats. Am J Vet Res 2018; 79: 181–190. 29359972 10.2460/ajvr.79.2.181

[116] Bian Z Jian X Liu G , et al. Wet-food diet promotes the recovery from surgery of castration and control of body weight in adult young cats. J Anim Sci 2023; 101. DOI: 10.1093/jas/skad039. PMC9997781 36734030

[117] Cameron KM Morris PJ Hackett RM , et al. The effects of increasing water content to reduce the energy density of the diet on body mass changes following caloric restriction in domestic cats. J Anim Physiol Anim Nutr 2011; 95: 399–408. 10.1111/j.1439-0396.2010.01107.x 21198957

[118] Schleicher M Cash SB Freeman LM . Determinants of pet food purchasing decisions. Can Vet J 2019; 60: 644–650. 31156266 PMC6515811

